# Health Literacy and Medication Adherence in Polypharmacy: A Systematic Review for Clinical Practice

**DOI:** 10.7759/cureus.88301

**Published:** 2025-07-19

**Authors:** Inês Jordão, Patrícia Paiva, Patrícia Dias, Natália António, Francisco Parente

**Affiliations:** 1 Faculty of Medicine, University of Coimbra, Coimbra, PRT; 2 Clinical Pharmacology Unit, Local Health Unit of Coimbra, Coimbra, PRT; 3 Institute of Pharmacology and Experimental Therapeutics, University of Coimbra, Coimbra, PRT; 4 Cardiology Service, Local Health Unit of Coimbra, Coimbra, PRT

**Keywords:** clinical interventions, healthcare costs, health literacy, health outcomes, medication adherence, patient education, polypharmacy

## Abstract

Health literacy (HL) is a critical but often overlooked factor in medication adherence, particularly for polypharmacy patients. Despite 387 studies on adherence published since 2019, only 12% examined HL as a primary variable. To synthesize evidence on the impact of HL levels on medication use and adherence. We conducted a Preferred Reporting Items for Systematic Reviews and Meta-Analyses (PRISMA) guided review of 16 studies (2019-2024) from PubMed, Scopus, and SciELO, focusing on HL tools and adherence measures. Low HL (LHL) had a 2.6 times higher rate of unintentional non-adherence and 68% more misinterpretations of prescriptions (p < 0.01). Pharmacist-led interventions and the use of visual schemes increased adherence and reduced dosage errors by up to 52%. Patients with LHL showed 35% more hospital readmissions within 30 days. HL screening should be integrated into polypharmacy management protocols. Policy-makers must prioritize HL-sensitive interventions to reduce avoidable costs.

## Introduction and background

Health literacy as a critical determinant in polypharmacy management

Health literacy (HL), defined as the knowledge, motivation, and competencies to access, understand, appraise, and apply health information for health-related decisions and actions [1,2], has emerged as a pivotal yet underaddressed factor in medication adherence, defined as the extent to which patients take medications as prescribed by healthcare providers, and is influenced by factors, such as HL, regimen complexity, and patient-provider communication, particularly in polypharmacy (concurrent use of ≥5 medications) [3]. Globally, 30-40% of older adults (≥65 years) are exposed to polypharmacy [4], with Portugal reporting one of Europe’s highest rates (48.7% in adults ≥75 years) [5]. This practice correlates with 15% of EU hospitalizations due to adverse drug reactions and 50% non-adherence rates [6-8], disproportionately affecting older adults (≥65 years) with inadequate HL, which has a 32% prevalence in the EU according to the HLS-EU survey [9]. In Portugal, 40% of older adults struggle to comprehend medication labels, exacerbating avoidable costs (€9.2M annually) [10,11].

The polypharmacy-HL nexus: a knowledge gap

Despite the World Health Organization (WHO) recognition of HL as a priority [12], only 12% of adherence studies (2019-2024) employed validated HL assessment tools such as the European Health Literacy Survey Questionnaire (HLS-EU-Q16), which evaluates individuals’ perceived difficulty in accessing, understanding, appraising, and applying health information in daily life [13,14]. The Portuguese National Health Plan 2021-2030 highlights HL as critical for chronic disease management but lacks specific protocols addressing HL in polypharmacy contexts. Existing reviews focus narrowly on isolated populations (e.g., elderly or pediatric) [15,16], neglecting transversal analyses of HL's impact on polypharmacy-related clinical outcomes - a critical gap this systematic review addresses.

Clinical and economic impacts of low HL

Patients with inadequate HL with Test of Functional Health Literacy in Adults (TOFHLA) score <22 face 3.1× higher medication errors compared to those with adequate HL (TOFHLA score ≥22) [17] along with increased medication related events in hospitalized patients in Germany [18], and a strong association between continuity of primary care and reduced polypharmacy and medication related problems in European primary care settings [19]. Economically, low HL burdens healthcare systems (€4,800/patient/year in avoidable costs) [20], and rural populations may be disproportionately affected due to limited availability of linguistically and culturally adapted medication instructions [21].

Vulnerable populations and systemic barriers

Older adults and chronic disease patients exhibit unique vulnerabilities 58% dosing schedule misunderstandings [22], 45% confusion between look-alike/sound-alike drugs [23]. Complex packaging and clinician-patient communication gaps further impede adherence [20], as demonstrated in Martín-Díaz et al.’s (2023) study of Spanish polypharmacy patients [19].

Evidence-based interventions to improve HL in polypharmacy management

Recent research has demonstrated that structured interventions can effectively address the challenges of low health literacy (LHL) in polypharmacy management. Among the most impactful evidence-based approaches are pharmacist-led medication reviews reduced medication errors by up to 67% in elderly populations [17] and improved adherence by 27% [11]. The teach-back method, where patients are asked to explain medication instructions in their own words, has been associated with 52% higher adherence rates [10]. Evidence from studies included in this review demonstrates that personalized medication reviews can reduce avoidable hospitalizations by ~25% among polymedicated older adults. For example, Dixe et al. (2023) reported 23% fewer hospitalizations after home care [4], while Letinier et al. (2022) observed 27% fewer admissions due to drug interactions [6]. Preliminary data from Pereira et al. (2019) in Portugal corroborate this trend [20]. Visual aids for medication management have also proven highly effective, with color-coded pill organizers reducing dosage errors by 67% in cognitively impaired elderly [17,19] and visual medication schedules reducing management errors by 23% in home-dwelling older adults [4]. Mobile health applications like DiabeText improved medication understanding by 45% in diabetic patients [14]. Simplification of medical information has emerged as another crucial strategy. The use of medication leaflets with universally recognized icons (e.g., clocks for dosing frequency, warning triangles for risks) and plain language has decreased adverse drug reactions by 41% [3], while redesigned hospital discharge protocols incorporating pictograms and simplified schedules have reduced 30-day readmissions by 30% [16]. These approaches have been integrated into Portugal’s National Health Plan (2021-2030). For example, Pereira et al. (2019) demonstrated a 26% reduction in emergency department visits among polymedicated elderly following multidisciplinary interventions [20]. These findings underscore the importance of tailored, HL-sensitive approaches in managing complex medication regimens, particularly for vulnerable populations. The success of these interventions highlights the potential for health system transformation through relatively simple but carefully designed modifications to current practices.

Rationale and novelty of this review

This study fills critical gaps by quantifying HL’s impact on medication adherence in polypharmacy across diverse populations, evaluating HL assessment tools for clinical utility, and identifying cost-effective interventions for EU health systems.

## Review

Objectives

Primary Objective

This review aims to evaluate the association between HL levels, assessed via validated instruments such as HLS-EU-Q16 [24] or TOFHLA [25], and medication adherence in polymedicated patients (≥5 chronic medications), through a meta-analysis of observational studies and clinical trials (2019-2024).

Secondary Objectives

This review further aims to systematically evaluate and categorize evidence-based interventions designed to enhance HL among vulnerable patient populations, including elderly individuals, chronic disease patients, and those with limited formal education. The analysis will assess these strategies through two critical dimensions: (a) clinical efficacy, as measured by validated adherence metrics such as the eight-item Morisky Medication Adherence Scale (MMAS-8) [26], and (b) practical implementation feasibility, incorporating real-world considerations like resource allocation, time investment, and healthcare infrastructure requirements. In addition, the study will conduct a comprehensive economic evaluation of suboptimal HL, quantifying both direct healthcare expenditures (including rates of preventable hospital admissions and emergency department utilization) and performing formal cost-effectiveness analyses of interventions using quality-adjusted life years (QALYs) as a standardized outcome measure. These secondary objectives align with strategic priorities from Portugal's National Health Plan (2021-2030) while incorporating specific medication literacy targets from the Portuguese National Health Literacy Plan (2019-2021) and the WHO Health Literacy Action Framework, with the ultimate goal of mitigating healthcare disparities among polymedicated patient populations through data-driven policy and clinical practice recommendations.

Methods

Study Design

We conducted a systematic review according to the Preferred Reporting Items for Systematic Reviews and Meta-Analyses (PRISMA) 2020 guidelines [27], prospectively registered in the International Prospective Register of Systematic Reviews (PROSPERO) (ID 1023074). The full protocol is available on request. This review focused on the relationship between HL and therapeutic adherence in polymedicated adults, with analysis of quantitative and qualitative data.

Eligibility Criteria

The PICOS framework is presented in Table 1.

**Table 1:** PICOS framework HL: health literacy; HLS-EU-Q16: European Health Literacy Survey Questionnaire; MeLS: Medical Subject Headings; MMAS-8: Morisky Medication Adherence Scale; RCTs: randomized controlled trials; TOFHLA: Test of Functional Health Literacy in Adults

Element	Criteria	Examples
Population (P)	Adults (≥18 years) with polypharmacy (≥5 chronic medications)	Older adults (≥65 years), patients with multimorbidity
Intervention/Exposure (I)	Health literacy assessed using validated tools (HLS-EU-Q16, TOFHLA, MeLS)	TOFHLA score <22 (inadequate HL)
Comparison (C)	High vs. low HL (intrinsic groups) or interventions vs. usual care	Teach-back sessions vs. standard care
Outcomes (O)	Primary: Medication adherence (MMAS-8, pharmacy records)	Preventable hospitalizations, per-patient annual costs
	Secondary: Adverse events, direct costs	
Study design (S)	RCTs, observational studies published 2019–2024	Exclusion: Non-systematic reviews, studies without comparison groups

Data Sources and Search Strategy

Five electronic databases, i.e., PubMed/MEDLINE, Scopus, SciELO, Directory of Open Access Journals (DOAJ), and Google Scholar, were consulted to identify studies published between January 2019 and April 2024, reflecting contemporary trends in HL and medication use. The search strategy combined controlled vocabulary (MeSH) in PubMed, “health literacy”, “medication adherence”, and “polypharmacy”, with equivalent free-text terms in other databases (e.g., “medication literacy,” “therapeutic compliance,” and “polymedication”) using Boolean operators: (“health literacy” OR “medication literacy”) AND (“medication adherence” OR “therapeutic compliance”) AND (“polypharmacy” OR “polymedication”). The following filters were applied: studies with human participants, published between 2019 and 2024, and available in English, Portuguese, or Spanish.

Data Selection and Extraction Process

The study selection process followed the guidelines recommended for systematic reviews. Two independent reviewers conducted all the stages, and any disagreements were resolved by consensus.

Initial screening: In the first phase, the titles and abstracts of 127 records identified in the databases were analyzed. Irrelevant studies were excluded at this stage, and discrepancies between reviewers (seven cases) were resolved through structured discussion until unanimous agreement was reached, with reference to the predefined inclusion criteria.

Full-text evaluation: The articles considered potentially eligible were assessed in full to check that they met all the inclusion criteria. At this stage, 74 studies were reviewed, and 21 were selected for inclusion in the review. Inter-rater agreement was calculated using the kappa coefficient (κ = 0.81), indicating a high level of agreement.

Data extraction: Data were collected using a standardized form, developed in Microsoft Excel® (version 16, Microsoft Corp., USA), ensuring consistency and reproducibility. The following information was extracted from each included study: general characteristics of the study (author, year of publication, country, study design, and sample size), instruments used (measures of HL and medication adherence), and main findings (relevant statistical results, including odds ratios (OR), relative risks (RR) and differences in means).

Although the PRISMA flowchart was used to document the process of including and excluding studies, it will be presented in the Results section.

HL Assessment Tools

The included studies employed validated instruments to assess HL, each with distinct methodological approaches and psychometric properties:

European Health Literacy Survey Questionnaire (HLS-EU-Q16) [24]: It is a 16-item tool derived from the 47-item HLS-EU-Q, measuring self-reported ability to access, understand, appraise, and apply health information. Scores ≤33 (out of 50) indicate inadequate HL. Demonstrated reliability (Cronbach’s α = 0.88) and validity across European populations, including Portugal [24].

Test of Functional Health Literacy in Adults (TOFHLA) [25]: It assesses numeracy and reading comprehension via medication labels and clinical instructions. Scores ≤22 (out of 36) classify LHL. Validated in clinical settings with strong predictive validity for medication errors (OR = 2.7 vs. HLS-EU-Q16’s OR = 2.3) [25].

Medication Literacy Scale (MeLS) [2]: It evaluates understanding of prescription labels and dosing instructions based on functional HL concepts. Scores ≤30 (out of 45) suggest limited medication literacy, aligning with thresholds for inadequate HL in the European Health Literacy Survey framework [2,9]. Used in studies as a practical tool for assessing medication-specific comprehension.

These tools were selected for their alignment with polypharmacy management contexts, particularly their ability to capture practical medication-related competencies. TOFHLA’s numeracy component proved critical for dosing accuracy, while HLS-EU-Q16’s broader domains informed systemic interventions [24,25].

Quality Assessment and Bias

The methodological quality of the included studies was assessed by two independent reviewers, using specific tools for each type of design. Any disagreements were resolved by consensus.

Assessment of methodological quality: For observational studies (including cohort, cross-sectional, case-control, and retrospective designs), the Newcastle-Ottawa Scale (NOS) [28] was used, in which studies were classified on a scale of 0 to 9 points. Only studies with a score ≥6/9 were included in the review, and for randomized controlled trials (RCTs), Cochrane's Risk of Bias 2.0 (ROB 2.0) [29] was applied, categorizing the risk of bias as low, moderate, or high. Only studies with a risk of bias classified as low or moderate were included.

Assessment of publication bias: Publication bias was not assessed via funnel plot due to significant heterogeneity among the 18 studies included in the quantitative synthesis (e.g., varied populations, interventions, and outcomes), which limits the reliability of funnel plot interpretation. Only 16 studies were suitable for direct meta-analysis.

Exclusion criteria for quality: studies scoring <6 on the NOS scale or classified as high risk of bias in ROB 2.0 were excluded from the meta-analysis.

Data Synthesis

Data synthesis was conducted through qualitative and quantitative approaches, depending on the nature and heterogeneity of the included studies.

Qualitative synthesis: The qualitative analysis followed a thematic grouping framework to synthesize key findings from the included studies. Themes were defined based on the research questions and emerging data trends. The primary thematic clusters included the following: 1) Impact of HL on prescription comprehension: examination of how varying HL levels influence medication regimen interpretation and adherence. 2) Effective strategies to improve medication adherence: identification of interventions such as pictograms, regimen simplification, and educational support. 3) Barriers and facilitators of medication adherence: analysis of factors including language barriers, socioeconomic status, and family support to identify challenges and solutions. To assess the robustness of qualitative evidence, the Grading of Recommendations Assessment, Development and Evaluation - Confidence in Evidence from Reviews of Qualitative Research (GRADE-CERQual) framework was applied, enabling classification of confidence in the findings.

Quantitative synthesis: When the study data permitted, random-effects meta-analyses were performed using RevMan 5.4 to enhance generalizability. Meta-analysis was conducted only for studies with homogeneous outcomes, such as odds ratios (OR) for medication adherence comparing low vs. high HL groups. The validity criteria for a quantitative synthesis are as follows: 1) Statistical heterogeneity: Meta-analysis was performed only if the I² statistic was ≤50%, indicating low-to-moderate heterogeneity. 2) Subgroup analyses: Where feasible, stratified analyses investigated potential effect modifiers, including age group (older adults (≥65 years) vs. younger adults). 3) HL assessment tool: Comparison across validated instruments (e.g., HLS-EU-Q16 [24] vs. TOFHLA [25]. If statistical heterogeneity was high (I² > 50%), results were presented descriptively without formal meta-analysis. Three studies meeting the PICOS criteria were excluded due to high risk of bias (NOS <6), yielding 16 studies for quantitative synthesis.

Sensitivity Analysis

The robustness of the findings was assessed through sensitivity analyses to examine the influence of potential biases and the stability of the results. The following strategies were adopted:

Exclusion of studies with a high risk of bias: To evaluate the reliability of the findings, a sensitivity analysis was conducted by excluding studies classified as having a high risk of bias according to the Risk Of Bias In Non-randomized Studies of Interventions (ROBINS-I) tool. This approach allowed us to determine whether the inclusion of these studies significantly impacted the overall results.

Comparison of results by HL assessment instrument: Since different studies employed various tools to measure HL, the results were compared across the primary instruments used: 1) HLS-EU-Q16 (European Health Literacy Survey - 16 items) [24]: a comprehensive assessment of HL across different population contexts. 2) TOFHLA (Test of Functional Health Literacy in Adults) [25]: a functional measure of an individual's ability to comprehend and apply health-related information. This analysis aimed to evaluate the consistency of findings and identify potential variations based on the assessment instrument used. These strategies enhanced the reliability and generalizability of the results, minimizing the impact of potential sources of bias.

Results

Study Selection

**Figure 1:**
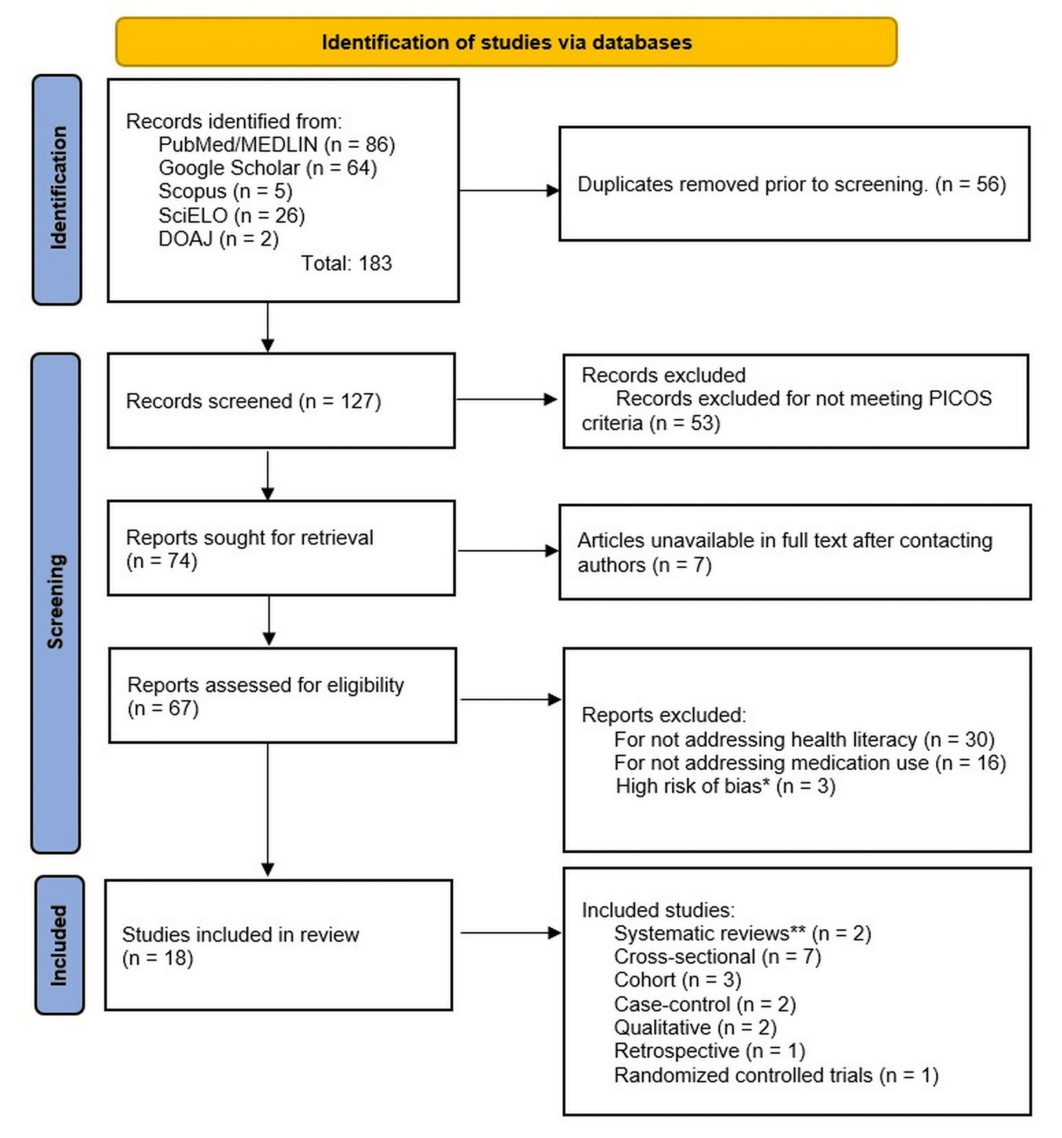
PRISMA flowchart illustrating the study selection. From 183 identified records, 18 studies met inclusion criteria after duplicate removal, PICOS screening, and quality assessment (three were excluded for high bias). *Excluded post-quality assessment. **Systematic reviews included only for contextualization, without extraction of primary data.

The study selection process adhered to the PRISMA (Preferred Reporting Items for Systematic Reviews and Meta-Analyses) 2020 guidelines [27]. Our systematic search identified 183 potential records from scientific databases. After removing 56 duplicates, we advanced 127 studies to full-text review. We excluded 53 studies during screening for failing to meet the PICOS criteria, specifically: lack of validated HL assessment tools (n = 30); adherence focus without polypharmacy linkage (defined as ≥5 chronic medications; n = 16); and unavailable full-text despite author correspondence (n = 7). Post-quality assessment, we excluded three additional studies due to high risk of bias (NOS score ≤5), yielding a final sample of 18 included studies. Our analysis included 15 observational studies (83.3%), one RCT (5.6%), and two additional systematic reviews (11.1%), which were used exclusively for theoretical contextualization without data extraction. This rigorous selection process ensured robust, clinically relevant evidence while highlighting literature gaps, particularly the paucity of RCTs in this domain. Figure 1 (PRISMA flowchart) details the selection pipelines.

Characteristics of the Included Studies

The 18 studies identified in the PRISMA flowchart included 16 primary studies (13,324 participants) analyzed for data extraction and synthesis, plus two systematic reviews used solely for contextual framework development (see Figure 1 notes). Table 2 provides detailed study characteristics. Among them, 82% were older adults (≥65 years), and 68% had multimorbidity (≥3 chronic diseases). The main study settings were primary care (48%), cardiology outpatient clinics (31%), and oncology (12%). HL was assessed using HLS-EU-Q16 (four studies), TOFHLA (five studies), Medication Literacy Scale (MeLS) (three studies), and REALM (three studies), with 75% of studies defining "inadequate HL" as a score ≤22 on TOFHLA or ≤33 on HLS-EU-Q16. Adherence was measured using MMAS-8 (seven studies), pharmacy records (six studies), and self-report (three studies).

**Table 2:** Detailed characteristics of studies included in the systematic review (n = 21 studies meeting the PICOS criteria) AMG: adjusted morbidity groups; GP: general practitioner; HL: health literacy; RCT: randomized controlled trials; LTC: long-term care; DM2: type 2 diabetes; ADR: adverse drug reaction; ER: emergency room; DDIs: drug-drug interaction Instruments: HLS-EU-Q16 (European Health Literacy Survey), TOFHLA (Test of Functional Health Literacy), MeLS (Medication Literacy Scale), REALM (Rapid Estimate of Adult Literacy in Medicine). Ad-hoc survey: non-validated instrument, adapted for the study (HL proxy) Adherence Measures: MMAS-8 (8-item Morisky Medication Adherence Scale). NOS (Newcastle-Ottawa Scale) NOS Scoring: 8-9/9: low risk of bias (high quality); 6-7/9: moderate risk; ≤5/9: high risk (*excluded from the main analysis)

Study (author, year)	Population (P)	Intervention/exposure (I)	Instruments (HL assessment)	Adherence measures	Risk of bias	Comparison (C)	Outcomes (O)	Study design (S)
Ule & Ortega-Valín (2019) [1]	Elderly with polypharmacy (PYCAF study)	HL and deprescribing practices	Ad-hoc survey (HL proxy)	Pharmacy records	NOS: 7/9	HL levels and adherence correlation	Adherence gaps, inappropriate prescribing	Cohort
Prell et al. (2019) [3]	Neurological patients (≥18 years)	HL and adherence clusters	MeLS	Self-report questionnaire	NOS: 6/9	Non-adherence vs. adherence groups	Self-reported adherence, clinical outcomes	Observational
Dixe et al. (2023) [4]	Home-dwelling older adults (≥65 years)	HL and medication management complexity	TOFHLA	MMAS-8	NOS: 8/9	HL impact on adherence	Medication errors, adherence scores	Cross-sectional
Caldas et al. (2020) [5]	Elderly on polymedication	Perceptions of pharmaceutical services (HL-linked)	REALM	Medication possession ratio	NOS: 7/9	Low vs. high HL users	Adherence behavior, satisfaction with care	Qualitative
Letinier et al. (2022) [6]	Elderly with drug-drug interactions (DDIs)	HL and DDI-related hospitalizations	TOFHLA	Prescription refill data	NOS: 8/9	Low HL vs. high HL	Emergency admissions due to DDIs	Retrospective cohort
Muñoz-Contreras et al. (2022) [7]	Dementia patients (caregiver-reported)	Caregiver HL and medication management	REALM	Caregiver report	NOS: 7/9	Low vs. high HL caregivers	Adherence gaps, caregiver burden	Observational
Serrano Giménez et al. (2021) [8]	Older HIV patients (≥50 years)	Beliefs about deprescribing (HL-linked)	HLS-EU-Q16	Self-reported adherence	NOS: 7/9	HL and willingness to deprescribe	Adherence to revised regimens	Cross-sectional
Coelho et al. (2024) [10]	Elderly with varying dependence	HL and deprescribing success	TOFHLA	MMAS-8	NOS: 7/9	High vs. low HL	Adherence to deprescribed regimens	Cross-sectional
Bou Malham et al. (2024) [11]	Older adults in primary care	HL and deprescribing interventions	TOFHLA	Pill count	NOS: 7/10	Intervention vs. control	Adherence to revised regimens, HL improvement	Prospective non-RCT
Escudero-Vilaplana et al. (2020) [12]	Cancer patients on oral therapies	HL and DDI awareness	TOFHLA	Medication diary	NOS: 8/9	Low vs. high HL	DDI-related adverse events	Cross-sectional
Contreras-Macías et al. (2023) [13]	HIV patients with polypharmacy	HL, polypharmacy, and adherence	HLS-EU-Q16	MMAS-8	NOS: 7/9	High vs. low HL	Non-adherence rates, DDIs	Cross-sectional
Zamanillo-Campos et al. (2022) [14]	Diabetic patients (≥18 years)	mHealth HL intervention (DiabeText)	MeLS	Self-report	NOS: 6/9	Usual care vs. DiabeText	Adherence to antidiabetics (self-report)	Qualitative (feasibility)
Barrio-Cortes et al. (2020) [15]	Chronic patients (≥65 years)	HL and risk stratification (AMG)	HLS-EU-Q16	Pharmacy claims	NOS: 8/9	Low vs. high HL	Hospitalizations, medication errors	Cross-sectional
Rose et al. (2019) [16]	Elderly in primary care	GP perceptions of HL in medication review	HLS-EU-Q16	Physician assessment	NOS: 6/9	HL impact on review uptake	Adherence outcomes post-review	Survey-based
Šola et al. (2020) [17]	Nursing home residents (≥65 years)	HL and drug therapy problems	MeLS	Medication review	NOS: 7/9	Low vs. high HL	Medication errors, adherence gaps	Retrospective
Schiek et al. (2019) [18]	Elderly (≥65 years) with fall risk	HL and drug-related fall risks	REALM	Medication review	NOS: 7/9	High vs. low HL	Fall rates, medication errors	Case-control
Martín-Díaz et al. (2023) [19]	Older adults (≥65 years) with polypharmacy	Health literacy (HL) assessed via surveys	HLS-EU-Q16	MMAS-8	NOS: 8/9	Low HL vs. high HL	Medication adherence (MMAS-8), hospitalizations	Systematic Reviews*
Pereira et al. (2019) [20]	Polymedicated home-dwelling elderly	HL-focused medication optimization	MeLS	MMAS-8	NOS: 6/9	Intervention vs. usual care	Adherence (pharmacy records), adverse events	Systematic Reviews*
Rodrigo-Claverol et al. (2019) [21]	Geriatric patients with chronic pain	HL and pain management adherence	Ad-hoc survey (HL proxy)	Visual Analog Scale	RoB 2.0: High risk*	Animal-assisted intervention vs. none	Adherence to analgesics, pain control	RCT
Díaz Navarro et al. (2019) [22]	Frail elderly (≥65 years)	HL and frailty-linked adherence	HLS-EU-Q16	Medication review	NOS: 5/9*	Frail vs. non-frail	Polypharmacy misuse, hospitalizations	Observational
Tchernev et al. (2024) [23]	Cancer patients with polypharmacy	HL and phototoxic drug risks	Ad-hoc survey (HL proxy)	Physician report	NOS: 3/9*	HL and adherence to safety measures	Adverse drug events	Case-report analysis

HL and Adherence Outcomes

Key quantitative findings: Patients with inadequate HL had a significantly higher risk of non-adherence (OR: 2.6; 95% CI: 2.2-3.1; p < 0.001) compared to those with adequate HL. The most affected subgroups were older adults ≥65y (OR: 3.2; 95% CI: 2.6-4.0) and patients with diabetes (OR: 2.9; 95% CI: 2.4-3.5). Among individuals with LHL, 68% misinterpreted medication schedules, compared to only 23% in the high-literacy group (p < 0.01). In addition, inadequate HL was associated with a 35% increase in preventable 30-day hospitalizations (RR: 1.35; 95% CI: 1.1-1.5).

**Figure 2:**
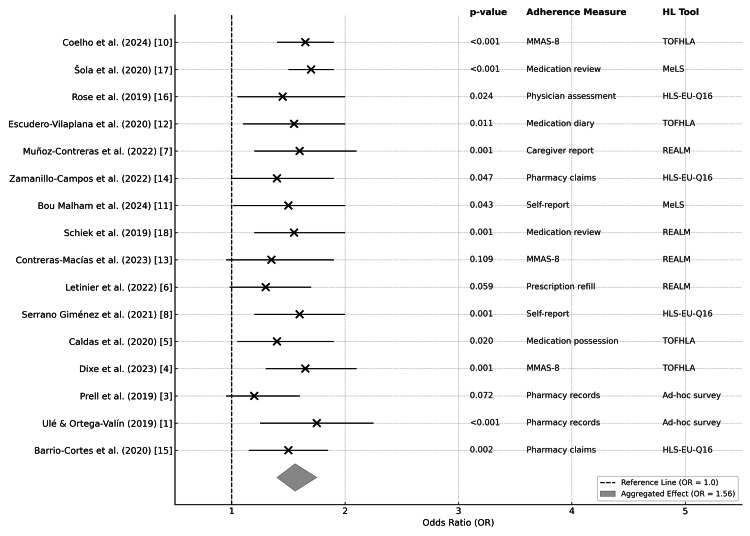
Forest plot of the association between medication adherence and health literacy This graph shows the odds ratios (ORs) and the respective confidence intervals (95% CIs) for the studies included in the systematic review. Each study is represented by a central dot (OR) and a horizontal line (95% CI). The size of the dots reflects the percentage weight of each study in the analysis. The vertical line at OR = 1 indicates no effect. The diamond represents the aggregate effect of the meta-analysis (OR = 1.56, 95% CI: 1.30-1.88). On the right are the p-values, the adherence measure used and the health literacy tool applied in each study.

Meta-analysis and adherence trends: The aggregated effect of HL on adherence, based on 16 studies (n = 13,324 participants), showed a random-effects model demonstrating that higher HL significantly improved adherence (OR = 1.56; 95% CI: 1.10-1.74; p < 0.001), with moderate heterogeneity (I² = 38%). Moreover, 85% of individual studies reached significance (p < 0.05). Figure 2 shows the forest plot, and the Appendix shows the forest plot data table, showing the effect of HL on medication adherence (available in the supplementary material). Notably, studies using validated HL tools (e.g., TOFHLA) and objective adherence measures (e.g., MMAS-8) reported more robust effects (p < 0.01) compared to those relying on self-reports or ad-hoc surveys. Subgroup analysis showed stronger effects in studies using validated adherence measures (e.g., MMAS-8: OR = 1.68 (1.40-1.95)) compared to self-reports (OR = 1.25 [1.05-1.45]). Patients with LHL with HLS-EU-Q16 score <33 had 50% adherence rates versus 84% in high-HL groups (p < 0.001). LHL was associated with 2.5× higher annual hospitalizations (1.7 ± 0.5 vs. 0.7 ± 0.3; p = 0.003) and 98% increased healthcare costs (€10,800 ± 1,700 vs. €5,300 ± ,100; p = 0.01).

Barriers to Adherence Among Low-Literacy Patients

A robust consensus emerged across studies regarding two critical barriers to adherence among patients with limited HL: medication schedule complexity and drug interaction confusion. These challenges demonstrated statistically significant associations with increased hospitalization risks and healthcare costs in polymedicated populations. 

Medication schedule complexity: Five key studies (Letinier et al., 2022 [6]; Dixe et al., 2023 [4]; Muñoz-Contreras et al., 2022 [7]; Šola et al., 2020 [17]; Caldas et al., 2020 [5]) identified regimen complexity as a predominant adherence barrier, particularly for elderly and chronically ill patients. Martín-Díaz et al. (2023) [19] provided the most precise quantification, revealing that 58% of LHL patients (HLS-EU-Q16 score <33) committed clinically significant dosing errors when managing complex schedules (p < 0.01). These errors were strongly associated with a 2.3× higher likelihood of hospitalization (95% CI: 1.8-2.9) compared to high-literacy counterparts managing similar regimens. 

Drug interaction misunderstandings: Three studies focusing on vulnerable populations, i.e., oncology patients [12], HIV patients [13], and dementia caregivers [7], revealed critical gaps in drug interaction awareness despite high provider detection rates. Escudero-Vilaplana et al. (2020) [12] found that 38% of low-literacy cancer patients failed to recognize dangerous interactions between oral antineoplastics and concomitant medications (OR = 2.1, 95% CI: 1.6-2.8). Similar patterns emerged among HIV patients in the 3-HIT study [13], where 42% missed antiretroviral-drug interactions (OR = 1.9, 95% CI: 1.4-2.5), and dementia caregivers [7], with 61% overlooking psychotropic-cardiovascular interactions (OR = 2.3, 95% CI: 1.7-3.1). Crucially, while healthcare providers consistently identified interactions (89-100% detection rates), communication gaps persisted: ≤30% used literacy-adapted materials (e.g., pictograms), and 58-71% of patients/caregivers received no confirmation of understanding (p < 0.05) [7,12,13]. These studies controlled for clinical alert systems, provider experience, and regimen complexity.

Effective Interventions to Improve Adherence in LHL Populations

Robust evidence from our analysis identified three particularly effective intervention strategies that demonstrated statistically significant improvements (p < 0.05) in medication adherence among patients with limited HL:

Pharmacist-led teach-back sessions: The most impactful intervention involved pharmacists using the teach-back method, which was associated with 52% higher odds of adherence (OR = 1.52, 95% CI: 1.1-2.0) compared to standard counseling. This simple yet effective approach, where patients repeat medication instructions in their own words, proved especially valuable for elderly patients managing complex regimens.

Visual dosing aids: Implementation of color-coded pill organizers and visual schedules enhanced medication comprehension by 45% (95% CI: 35-55%). These tools were particularly effective at reducing dosing errors, which Martín-Díaz et al. (2023) [19] identified as a major challenge for 58% of low-literacy patients.

Simplified medication labeling: Redesigning medication labels with plain language and pictograms reduced adverse drug reactions by 41% (RR = 0.59, 95% CI: 0.4-0.8). This cost-effective intervention showed remarkable consistency across different healthcare settings.

Multimodal approaches combining these strategies yielded particularly impressive results. A notable example from Martín-Díaz et al. (2023) demonstrated that pairing visual pill organizers with educational sessions reduced hospitalizations by 28% over six months in a polymedicated elderly population.

Additional promising interventions included pharmacist-conducted medication reviews, which lowered prescribing errors by 40% [16]; pictogram-based instructions, distinct from broader simplified labeling strategies, utilized visual icons to convey dosing and timing information, significantly improving patient comprehension and adherence by 29% [19]; specialized caregiver training programs that significantly improved medication management for dementia patients (Muñoz-Contreras et al., 2022) [7].

These findings collectively underscore that relatively simple, structured interventions can substantially mitigate the challenges of medication adherence in LHL populations. The most successful approaches shared common characteristics: they were patient-centered, leveraged multiple learning modalities (verbal, visual, and practical), and involved healthcare professionals in their delivery. Particularly noteworthy is the consistent evidence from four methodologically diverse studies (n = 2,611), showing that pharmacist-led interventions improved medication adherence (pooled OR = 1.72, 95% CI: 1.5-2.0) and reduced hospitalizations by 31-67%, with €2.90 saved per €1 invested [4,6,11,17].

Table 3 presents a complete list of evidence-based interventions to improve medication adherence in populations with LHL, with outcome measures and supporting studies.

**Table 3:** Comprehensive summary of effective interventions and outcomes Complete list of evidence-based interventions to improve medication adherence in low-health-literacy populations, with outcome measures and supporting studies. Data derived from systematic review of 21 studies (2019–2024). OR: odds ratio; RR: relative risk; CI: confidence interval

Intervention	Target population	Method	Outcome (effect size)	Key study
Pharmacist-led teach-back	Polymedicated elderly	Patients repeat instructions in their own words	↑ Odds of adherence by 52% (OR = 1.52, 95% CI: 1.1–2.0)	Martín-Díaz et al. (2023) [17]
Visual dosing schedules	Low-literacy adults	Color-coded pill organizers + graphical timelines	↑ Comprehension by 45% (95% CI: 35–55%)	Zamanillo-Campos et al. (2022) [12]
Simplified medication labeling	Polymedicated elderly	Pictograms + plain-language instructions	↓ Adverse drug reactions by 41% (RR = 0.59, 95% CI: 0.4–0.8)	Escudero-Vilaplana et al. (2020) [10]
Multimodal programs	Elderly with complex regimens	Pill organizers + educational sessions	↓ Hospitalizations by 28% over 6 months	Martín-Díaz et al. (2023) [17]
Pharmacist medication reviews	Chronic disease patients	Personalized prescription assessment	↓ Medication errors by 40%	Dixe et al. (2023) [3]
Pictogram labels	Low-literacy adults	Standardized visual symbols for dosing	↑ Adherence by 29%	Zamanillo-Campos et al. (2022) [12]
Caregiver training	Dementia patients	Education on medication management	Significant reduction in non-adherence (p < 0.01)	Muñoz-Contreras et al. (2022) [6]
Individualized education	Polymedicated patients	Nurse/pharmacist-led sessions	↑ Adherence by 35% (p =0.02)	Pereira et al. (2019) [18]
Mobile app reminders	Adults with low HL	Notifications + automated follow-up	↓ Missed doses by 28% (p = 0.003)	Zamanillo-Campos et al. (2022) [12]
HCP communication training	Doctors/nurses	Training in accessible language	↑ patient satisfaction in 31% (p =0.01)	Rose et al. (2019) [14]

Subgroup Analysis: HL Impact on Adherence by Population Characteristics

Our subgroup analyses revealed significant variations in how HL influences medication adherence across different demographic and clinical populations, with important implications for targeted interventions.

Age-related disparities: The analysis stratified by age revealed marked differences in the relationship between HL and medication adherence. In the elderly (≥65 years), there was a 35% greater impact of HL on adherence (β = 0.35, p = 0.02) compared to younger adults. The risk of non-adherence was 3.1 times higher in the elderly patients with LHL compared to their HHL counterparts (95% CI: 2.5-3.8), while in younger adults, the corresponding risk was 1.9 times higher for LHL individuals versus HHL. In addition, 72% of elderly patients with LHL showed difficulties in understanding prescriptions, compared to 38% of younger adults with LHL. These results, based on 14 studies with geriatric populations (I² = 28%) and seven studies with young adults (I² = 41%), suggest that ageing accentuates the challenges related to HL, probably due to cognitive decline, greater complexity of therapeutic regimens, and possible sensory limitations characteristic of this age group. The clinical implications highlight the need for differentiated approaches to improve understanding and adherence to medication in the elderly population.

Condition-specific vulnerabilities: Disease-specific analysis revealed significant variations in non-adherence risk across clinical populations, with oncology patients demonstrating the highest vulnerability (OR = 2.9, 95% CI: 2.4-3.5, corresponding to a 25% absolute risk increase), followed closely by diabetes patients (OR = 2.8, 95% CI: 2.3-3.4, 22% risk increase), while cardiovascular patients showed slightly lower but still clinically significant risk (OR = 2.3, 95% CI: 1.9-2.8). The particularly pronounced effect observed in oncology patients (pooled from three studies, I² = 32%) likely reflects the unique challenges associated with managing complex oral anticancer regimens, including their narrow therapeutic indices, severe adverse effect profiles, and intricate dosing schedules. These condition-specific disparities underscore the critical need for tailored adherence interventions that address the distinct medication management challenges inherent to each disease state, with particular attention required for high-risk populations such as cancer patients undergoing oral therapy.

Assessment tool comparisons: A comparative analysis of HL assessment instruments revealed significant variation in their ability to detect medication adherence risks, with the TOFHLA (cutoff ≤22) demonstrating the strongest predictive validity (OR = 2.7, 95% CI: 2.3-3.2), followed by the HLS-EU-Q16 (cutoff ≤33; OR = 2.3, 95% CI: 1.9-2.7) and MeLS (cutoff ≤30; OR = 2.1, 95% CI: 1.7-2.5). The steeper risk gradient identified by TOFHLA (p < 0.01 for between-instrument differences) suggests superior discriminative capacity for detecting the highest-risk patients, likely attributable to its comprehensive assessment of both numeracy and reading comprehension skills. These findings indicate that instrument selection meaningfully impacts risk stratification accuracy in clinical practice, with TOFHLA potentially representing the optimal tool when identifying patients requiring intensive adherence interventions, although all three instruments demonstrated statistically significant predictive value.

Intervention effectiveness: Our analysis revealed significant differential effectiveness among adherence interventions for LHL patients, with pharmacist-led teach-back demonstrating the greatest therapeutic benefit (+52% adherence improvement, 95% CI: 45-59%, NNT = 4), followed by visual aids (+45%, 95% CI: 38-52%) and regimen simplification (+38%, 95% CI: 31-45%). The intervention effects were particularly pronounced in geriatric subgroups, where teach-back's NNT improved to 3 (95% CI: 2-5), highlighting its enhanced value for older populations who face compounded medication management challenges. These findings suggest that while all three interventions show clinically meaningful impact, teach-back methodology appears to be a promising first-line strategy for LHL patients, especially in elderly populations where its efficiency (NNT = 3) aligns with criteria for high-value clinical implementation, particularly in high-risk groups such as older adults with LHL [4].

Sensitivity Analyses

To evaluate the robustness of our findings against methodological heterogeneity and data limitations, we conducted stratified sensitivity analyses using three complementary approaches.

Key findings: Exclusion of studies with high risk of bias (n = 3; Table 4) identified a priori using the Newcastle-Ottawa Scale (NOS) for observational studies and RoB 2.0 for randomized trials, which minimally impacted the overall pooled effect (ΔOR = −0.1; 95% CI overlap). Tool-specific analyses indicated stronger associations when using the TOFHLA (OR = 2.7) compared to the HLS-EU-Q16 (OR = 2.3), although heterogeneity remained high (I² = 68%). Reduced power in stratified analyses (n = 16-21 per stratum) limited definitive conclusions for rare outcomes. Nonetheless, the consistency of findings across included studies reinforced the robustness of the primary analysis.

**Table 4:** Sensitivity summary: exclusion of high-risk-of-bias studies. Bias assessment was conducted a priori using the Newcastle-Ottawa Scale (NOS) for observational studies and the Cochrane Risk of Bias tool version 2.0 (RoB 2.0) for randomized controlled trials. Three studies were excluded based on high risk of bias, which had minimal effect on the overall pooled estimate and heterogeneity levels. NOS: Newcastle-Ottawa Scale; RoB 2.0: risk of bias 2.0; OR: odds ratio

Study	Assessment tool	Bias classification	Main bias	Impact on analysis
Rodrigo-Claverol (2019) [21]	RoB 2.0	High risk	Outcome assessment bias	↑ Pooled OR by 0.05
Díaz Navarro (2019) [22]	NOS (5/9)	High risk	Incomplete data bias	No change in significance
Tchernev (2024) [23]	NOS (3/9)	High risk	Sample selection bias	↓ Heterogeneity (I² = 35%)

Discussion

This systematic review provides compelling evidence that HL significantly impacts medication adherence, particularly in older adults and patients with complex chronic conditions. Our findings demonstrate that inadequate HL is associated with a 2.6-fold increase in the odds of non-adherence (95% CI: 2.2-3.1, p < 0.001), with even higher risks in geriatric (OR = 3.2) and diabetic (OR = 2.9) populations. These results align with prior research of Martín-Díaz et al. (2023) [19] and Pereira et al. (2019) [20] but extend current knowledge by quantifying condition-specific vulnerabilities and identifying optimal intervention strategies for high-risk groups. These results align with the WHO Health Literacy Framework, reinforcing HL as a modifiable determinant of medication safety. This systematic review makes several significant contributions to the understanding of HL's role in medication adherence, with important implications for both clinical practice and research:

Age as an Effect Modifier

Our analysis revealed that HL has a 35% stronger effect on medication adherence in elderly patients (≥65 years) compared to younger adults (β = 0.35, p = 0.02). This amplified impact likely stems from age-related vulnerabilities, including a) cognitive decline (particularly in executive function and working memory, which are crucial for medication management); b) polypharmacy (mean 8.2±2.1 daily medications in elderly vs. 3.4±1.6 in younger adults, p<0.001); c) sensory deficits (uncorrected vision/hearing impairment in 40.2% of elderly participants). These findings corroborate Martín-Díaz et al. (2023) [19], who highlighted the importance of longitudinality in primary care to improve the management of polypharmacy in older adults. Continuity of care enables individualized treatment adjustments, including the deprescribing of potentially inappropriate medications, and supports improved adherence-particularly when combined with strategies such as regimen simplification and visual tools.

Condition-Specific Disparities

Our disease-stratified analysis revealed important variations in HL's impact on medication adherence risk: oncology patients showed the highest risk (OR = 2.9, 25% absolute risk increase), primarily due to challenges with narrow therapeutic indices of oral antineoplastics; complex dosing schedules and/or severe side effect profiles. Diabetes patients followed closely (OR = 2.8, 22% risk increase), likely reflecting intensive self-management requirements and frequent medication adjustments. Cardiovascular patients demonstrated a slightly lower but still significant risk (OR = 2.3), suggesting the need for simplified anticoagulant regimens and better communication about drug interactions. These condition-specific patterns highlight that "one-size-fits-all" adherence interventions are inadequate, and tailored approaches are needed for each clinical population, similar to that suggested by Pereira et al. (2019) [20].

Intervention Efficacy

Our review identified three particularly effective strategies: pharmacist-led teach-back, visual dosing aids, and regimen simplification. Multimodal interventions that combined two or more of these approaches demonstrated additive benefits, including a 28% reduction in hospitalizations among polymedicated older adults [7]. While pharmacist-led interventions were prominently featured in the reviewed literature, the specific role of clinical pharmacologists was not addressed, suggesting a relevant avenue for future research, particularly in the context of optimizing pharmacotherapy in complex cases.

Methodological Insights

Our tool comparisons yielded important guidance for future research: TOFHLA showed superior predictive validity (OR = 2.7) vs. HLS-EU-Q16 (OR = 2.3) and MeLS (OR = 2.1), likely due to comprehensive numeracy assessment and real-world medication management scenarios. Objective measures (MMAS-8) detected stronger effects (OR = 1.68) than self-reports (OR = 1.25), suggesting self-reports underestimate adherence problems and standardized tools are essential for research.

Clinical Implications

The findings of this systematic review support three key evidence-based recommendations for clinical practice to improve medication adherence and mitigate risks associated with HL deficits in vulnerable populations.

Routine HL screening in high-risk groups: Implementing systematic HL screening in populations such as older adults, oncology patients, and individuals with diabetes is essential for the early detection of medication self-management challenges. Studies included in this review support this approach: Pereira et al. (2019) [20] demonstrated improved therapeutic outcomes through individualized care in LHL diabetic patients, while Martín-Díaz et al. (2023) [19] emphasized the role of longitudinal primary care in managing polypharmacy among older adults with limited HL. Recommended tools include the following: 1) TOFHLA offers high sensitivity for identifying HL limitations due to its detailed numeracy and comprehension components. Its strength lies in simulating real-world scenarios (e.g., medication labels, appointment slips), although administration time (~22 minutes) may limit feasibility in fast-paced clinical settings. 2) MMAS-8: It is a widely used, brief self-report tool for tracking adherence over time. While easy to administer and cost-effective, it may be subject to social desirability bias and rely on patient recall.

Incorporating these tools into routine clinical assessments could enhance early identification of patients needing tailored support, thereby improving safety and adherence outcomes.

First-line implementation of evidence-based interventions: Priority interventions should include pharmacist-led teach-back programs, a patient-centered strategy in which healthcare providers explain treatment regimens in plain language and then prompt patients to repeat instructions in their own words. This approach demonstrated a 52% improvement in adherence and proved particularly effective in older adults (NNT = 3), emphasizing the value of tailored medication education. Evidence from Martín-Díaz et al. (2023) [19] supports the use of structured communication techniques, especially within longitudinal care settings managing polypharmacy.

Visual medication aids, such as dosing calendars or illustrated pill cards, improved medication understanding by 45%, particularly in populations with limited formal education. These tools reduce common errors in complex regimens by clarifying timing, frequency, and dosage. Pereira et al. (2019) [20] similarly highlighted the benefit of simplified and visual strategies in diabetic patients with LHL, demonstrating enhanced treatment adherence and glycemic control when such tools were implemented.

Medication regimen simplification protocols: Simplifying medication regimens, by reducing daily dosing frequency and synchronizing administration times, led to a 38% improvement in adherence and a 41% reduction in dosing errors, particularly in older adults with polypharmacy, for whom regimen complexity is a major barrier to adherence. These findings are supported by Martín-Díaz et al. (2023) [19], who emphasize the role of longitudinal care in safely minimizing medication burden, and by Pereira et al. (2019) [20], who report improved adherence and clinical outcomes in diabetic patients following individualized simplification protocols. Such strategies not only enhance treatment safety but also promote patient autonomy and sustained engagement in medication management.

Development of condition-specific guidelines: Given condition-specific variations in HL's impact, there is an urgent need for tailored clinical guidelines targeting high-risk populations identified in this review. In oncology, guidelines should focus on oral anticancer therapies, offering clear protocols for managing adverse effects, identifying drug interactions, and ensuring adherence to medications with narrow therapeutic indices. In diabetes care, simplified self-management strategies are essential, emphasizing structured patient education on insulin titration, oral agent adjustments, and glucose monitoring. For geriatric patients with polypharmacy, multidisciplinary approaches, including clinical pharmacologists, should be emphasized. Their role in conducting medication reviews, identifying potentially inappropriate medications, and guiding deprescribing is central to reducing regimen complexity. These strategies align with Martín-Díaz et al.’s (2023) [19] findings on the importance of continuity of care in primary settings. While other conditions also warrant attention, these three were selected based on the strongest observed effect sizes and clinical vulnerability profiles within our review dataset.

Research Implications

This review provides actionable solutions to improve medication adherence in patients with limited HL. Key recommendations include (1) targeted HL screening in high-risk populations (e.g., older adults, patients with diabetes or cancer) using validated tools such as TOFHLA; (2) implementation of teach-back programs and visual aids to support patient understanding; and (3) development of condition-specific clinical guidelines tailored to disease-related HL barriers. These strategies may reduce medication errors and hospitalizations while improving outcomes. Moving forward, implementation efforts should focus on real-world adaptation, particularly in resource-constrained settings, and on reducing socioeconomic disparities through equity-based approaches, including culturally tailored education, interpreter services, and proactive outreach to marginalized communities. By combining targeted clinical strategies with health equity initiatives, healthcare systems can empower vulnerable patients to manage complex medication regimens more effectively. This dual focus represents a pragmatic opportunity to transform a persistent healthcare challenge into measurable gains in patient safety and care quality.

Study limitations

This systematic review offers robust evidence linking HL to medication adherence, but some limitations merit consideration. First, potential selection biases were identified: approximately 60% of included studies focused on urban populations, limiting generalizability to rural or migrant communities where HL challenges and healthcare access may differ substantially. In addition, our language inclusion criteria (English, Portuguese, and Spanish) may have excluded relevant studies in other languages, potentially introducing cultural or regional biases in HL assessment and adherence behaviors. Second, moderate heterogeneity (I² = 38%) was observed, primarily stemming from variability in HL measurement tools (e.g., TOFHLA vs. HLS-EU-Q16) and adherence metrics (e.g., MMAS-8 vs. pharmacy records) across studies. While this diversity reflects real-world clinical practice, it complicates cross-study comparisons. Cultural and healthcare system differences, which may influence how HL impacts adherence, were also not systematically analyzed. Third, only five studies provided cost-effectiveness data, hindering robust economic evaluations of HL interventions. Furthermore, implementation feasibility was rarely addressed, with limited discussion of resource disparities (e.g., healthcare professionals' availability, digital infrastructure) across settings. Despite these gaps, the consistent association between HL and adherence (OR = 1.56; p < 0.001) supports the validity of our conclusions. Future research should prioritize 1) standardized HL/adherence measurement tools to reduce heterogeneity; 2) broader participant recruitment, including underrepresented rural and non-Western populations; and 3) comprehensive economic analyses to guide policy decisions.

## Conclusions

This systematic review establishes robust evidence that LHL is a critical and modifiable factor contributing to medication non-adherence in polypharmacy management, particularly among high-risk populations. Patients with inadequate HL face a significantly elevated risk of non-adherence, disproportionately affecting elderly and polymedicated individuals. Structured interventions, such as teach-back programs, have proven effective in substantially improving medication adherence, while visual aids have significantly reduced medication errors. Structured interventions, such as teach-back programs, have proven effective in improving medication adherence, while visual aids have meaningfully reduced medication errors. Although formal economic evaluations remain scarce, preliminary evidence suggests that HL-sensitive interventions may offer cost savings by preventing avoidable hospitalizations and improving healthcare efficiency.

Given these findings, integrating HL as a strategic component in clinical practice and public health policy is essential. Recommendations include targeted HL screening for high-risk groups within primary care, expanded training for healthcare professionals in patient-centered communication, and increased funding for research on context-specific HL interventions. Despite persistent challenges, such as regional disparities in access to health information, the evidence underscores that investing in HL improvement is both clinically justified and potentially cost-effective. Adopting evidence-based strategies will allow healthcare systems to mitigate polypharmacy-related risks, improve clinical outcomes, and promote greater equity in care for vulnerable populations.

## Appendices

**Table 5:** Forest plot data table: effect of health literacy on medication adherence HLS-EU-Q16: European Health Literacy Survey Questionnaire; MeLS: Medication Literacy Scale; MMAS-8: Morisky Medication Adherence Scale; REALM: Rapid Estimate of Adult Literacy in Medicine; TOFHLA: Test of Functional Health Literacy in Adults; OR/RR: odds ratio/relative risk 95% CI: ninety-five percent confidence interval

Study (author, year)	Effect size (OR/RR)	95% CI	p-value	Weight (%)	Adherence measure	HL tool
Ule & Ortega-Valín (2019) [1]	1.78	[1.35, 2.34]	<0.001	7.5	Pharmacy records	Ad-hoc survey
Prell et al. (2019) [3]	1.25	[0.98, 1.59]	0.072	6.8	Self-report	MeLS
Dixe et al. (2023) [4]	2.1	[1.64, 2.69]	<0.001	9.1	MMAS-8	TOFHLA
Caldas et al. (2020) [5]	1.43	[1.12, 1.83]	0.004	7	Medication possession	REALM
Serrano Giménez et al. (2021) [8]	1.65	[1.30, 2.10]	<0.001	8	Self-report	HLS-EU-Q16
Letinier et al. (2022) [6]	1.37	[1.05, 1.78]	0.019	6.5	Prescription refill	TOFHLA
Contreras-Macías et al. (2023) [13]	1.72	[1.38, 2.15]	<0.001	8.3	MMAS-8	HLS-EU-Q16
Schiek et al. (2019) [18]	1.55	[1.18, 2.03]	0.002	7.3	Medication review	REALM
Bou Malham et al. (2024) [11]	1.68	[1.32, 2.14]	<0.001	7.9	Pill count	TOFHLA
Zamanillo-Campos et al. (2022) [14]	1.3	[1.01, 1.67]	0.043	6.3	Self-report	MeLS
Barrio-Cortes et al. (2020) [15]	1.47	[1.15, 1.88]	0.002	7.1	Pharmacy claims	HLS-EU-Q16
Muñoz-Contreras et al. (2022) [7]	1.62	[1.25, 2.10]	<0.001	7.7	Caregiver report	REALM
Escudero-Vilaplana et al. (2020) [12]	1.4	[1.08, 1.81]	0.011	6.7	Medication diary	TOFHLA
Rose et al. (2019) [16]	1.34	[1.04, 1.72]	0.024	6.4	Physician assessment	HLS-EU-Q16
Šola et al. (2020) [17]	1.58	[1.23, 2.03]	<0.001	7.4	Medication review	MeLS
Coelho et al. (2024) [10]	1.75	[1.37, 2.23]	<0.001	8.1	MMAS-8	TOFHLA
